# Transient Charging of Mixed Ionic‐Electronic Conductors by Anomalous Diffusion

**DOI:** 10.1002/adma.202507739

**Published:** 2025-08-23

**Authors:** Heyi Zhang, Gonzalo Rivera‐Sierra, Shirin Siahjani‐Gultekin, Jenifer Rubio‐Magnieto, Anis Allagui, Ignacio Sanjuán, David Franco, Antonio Guerrero, Enrique H. Balaguera, Juan Bisquert

**Affiliations:** ^1^ Instituto de Tecnología Química (ITQ) Universitat Politècnica de València‐ Consejo Superior de Investigaciones Científicas (UPV‐CSIC) València 46022 Spain; ^2^ Institute of Advanced Materials (INAM) Universitat Jaume I Castelló 12006 Spain; ^3^ Solar Energy Institute Ege University Izmir 35100 Türkiye; ^4^ Department of Sustainable and Renewable Energy Engineering University of Sharjah Sharjah PO Box 27272 UAE; ^5^ Escuela Superior de Ciencias Experimentales y Tecnología (ESCET) Universidad Rey Juan Carlos Móstoles Madrid 28933 Spain

**Keywords:** anomalous diffusion, impedance spectroscopy, mixed ionic‐electronic conductors, organic electrochemical transistors

## Abstract

Mixed ionic‐electronic conductors (MIECs) play a pivotal role in energy storage, bioelectronics, and neuromorphic computing. Understanding charge transport dynamics in these materials is crucial for optimising device performance. This study investigates the transient charging behavior of three representative MIEC systems: PEDOT:PSS, electrochromic WO_3_, and n‐doped PBDF polymer films via electrochemical impedance spectroscopy (EIS) and transient current measurements, focusing on anomalous diffusion. By employing the transmission line model and fractional exponent fitting, a strong correlation is revealed between impedance response and transient current dynamics. The findings provide insights into the role of fractional diffusion in MIEC charge transport and inform the design of advanced electrochemical devices.

## Graphical abstract

This article explores charge transport in mixed ionic‐electronic conductors (MIECs) through electrochemical impedance spectroscopy and transient current analysis. Focusing on PEDOT:PSS, WO_3_, and n‐doped PBDF, it uncovers the impact of anomalous diffusion via fractional modeling. The study reveals key correlations that deepen understanding and guide the design of next‐generation electrochemical devices.

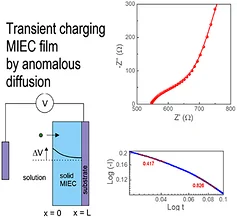

## 1 Introduction

Mixed ionic‐electronic conductors (MIECs) are key materials in a broad range of technologies, including lithium batteries,^[^ ^1^, ^2^ ^]^ solar energy materials,^[^ ^3^ ^]^ conducting polymer films,^[^ ^4^, ^5^ ^]^ electrochromic inorganic films,^[^ ^6^, ^7^ ^]^ and stimulation electrodes for biomedical devices.^[^ ^8^, ^9^ ^]^ In practical devices, a typical architecture involves a MIEC film interfaced with an external electrolyte, where ionic species are inserted electrochemically in response to an applied voltage. Among them, Poly(3,4‐ethylenedioxythiophene):polystyrene sulfonate (PEDOT:PSS) is a widely studied p‐type MIEC, offering a benchmark platform due to its high electronic conductivity, mechanical flexibility, and ionic permeability. It serves as the model system in numerous electrochemical and bioelectronic applications.^[^ ^10^, ^11^ ^]^ In parallel, inorganic materials such as amorphous tungsten trioxide (WO_3_) represent classical electrochromic systems exhibiting mixed conduction behavior and well‐defined reversible ion‐insertion mechanisms under electrochemical bias, making them ideal for fundamental studies of charge transport and storage.^[^ ^7^, ^12^ ^]^ Recently, n‐type conducting polymers such as n‐doped poly(benzodifurandione) (n‐PBDF) have gained growing attention as air‐stable, highly conductive n‐type MIECs, complementing their p‐type counterparts and enabling ambipolar organic electronics, electrophysiological monitoring, and OECTs.^[^ ^10^, ^11^, ^13^, ^14^ ^]^ These three representative systems—PEDOT:PSS, WO_3_, and n‐PBDF—together span a wide range of chemical structures, charge carrier types, and ion‐electron coupling strengths, forming a comprehensive material landscape for the design and mechanistic understanding of MIEC‐based devices.

In these systems, a MIEC film deposited on a conductive substrate is charged by the insertion of mobile ions from an external electrolyte, in a process that regulates electrochemically the charge storage and electronic conductivity of the film. The response of MIECs to an applied potential is governed by ion diffusion and charge redistribution processes, which directly affect device performance in terms of switching speed, stability, and overall efficiency. Diffusion charging is therefore a central dynamical aspect of MIEC‐based device operation, and it has important implications for the transient kinetics of organic electrochemical transistors (OECT).^[^ ^15^, ^16^, ^17^, ^18^ ^]^ The diffusion parameters of MIEC‐based devices are usually determined from electrochemical impedance spectroscopy (EIS) measurements. There is indeed a well‐known correlation between the impedance patterns of restricted diffusion^[^ ^19^, ^20^, ^21^ ^]^ and the transient current for a voltage step excitation ^[^ ^22^, ^23^, ^24^ ^]^ that is well described for ordinary diffusion.^[^ ^25^ ^]^ However, many electrochemical systems exhibit deviations from normal diffusion, leading to anomalous diffusion behavior characterized by fractional exponents.^[^ ^26^, ^27^, ^28^, ^29^, ^30^ ^]^

EIS and chronoamperometry (CA) are powerful techniques to probe the dynamic behavior of MIECs in the frequency and time domains, respectively. While both methods have been extensively used independently, a direct and quantitative correlation between EIS and CA data under anomalous diffusion has rarely been established. Theoretical models based on fractional calculus have proposed that anomalous diffusion yields specific impedance signatures and time‐domain power‐law decays, yet comprehensive experimental validation across multiple materials has been lacking.

**Figure 1:**
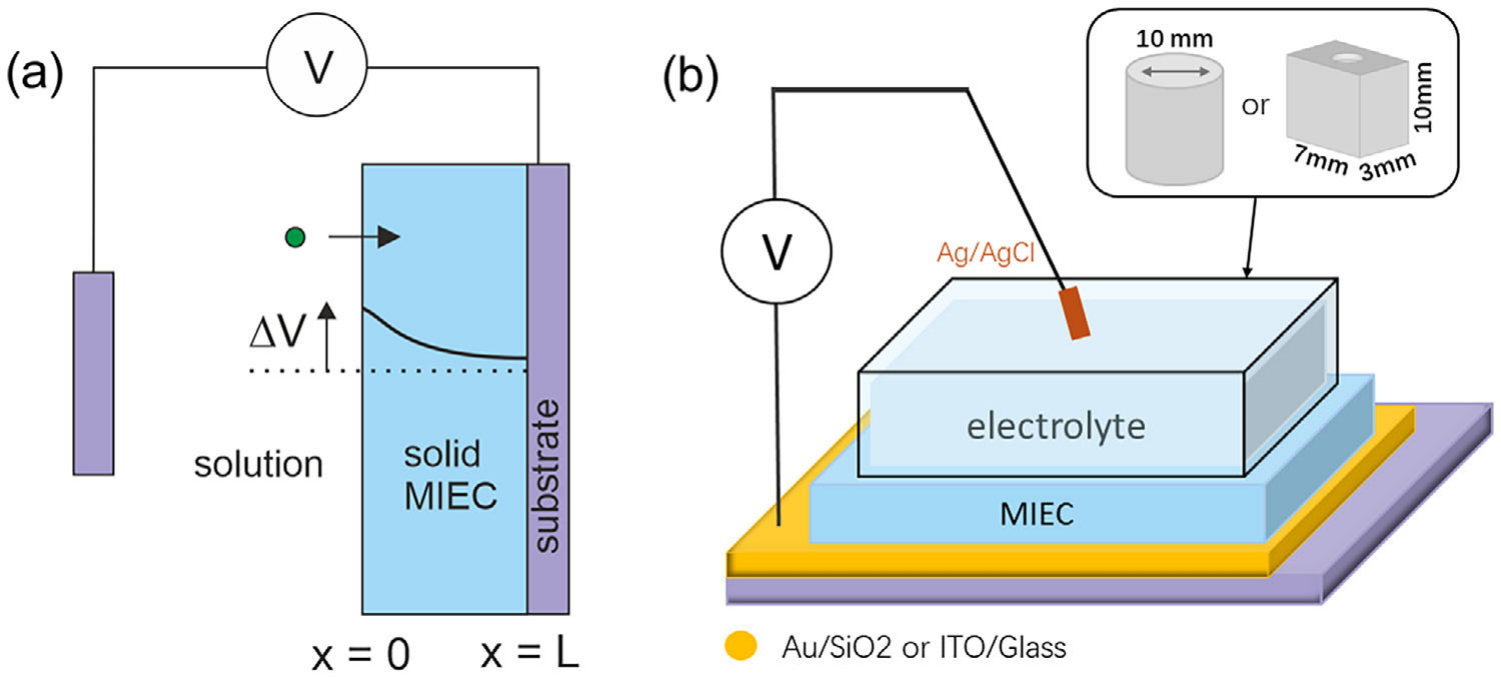
a) Scheme of the electrochemical insertion into a film of thickness *L*. b) Experimental setup for measurement of the MIEC‐electrolyte system.

In this work, we establish the full correspondence between frequency and time domain experiments in those electrochemical insertion experiments, as shown in **Figure** **1**, where anomalous diffusion with a fractional exponent occurs. We aim to obtain the frequency and time domain responses for the insertion of carriers by a small perturbation in a bounded domain in a region 0 < *x* < *L*.

The impedance corresponding to anomalous diffusion dynamics was already proposed by Bisquert and Compte ^[^ ^31^ ^]^, and it has been widely used in the interpretation of experiments involving ion diffusion in complex media.^[^ ^4^, ^5^, ^7^, ^32^, ^33^ ^]^

We first present a general theory that correlates the time and frequency domain responses with the respective power law limit. We combine time‐domain and frequency‐domain analyses to explore ion transport behavior under anomalous diffusion conditions. Specifically, we apply the fractional transmission line model to interpret impedance results and use a fractional power‐law decay model to describe the transient current response following a voltage step. We then experimentally validate this model across three distinct MIEC systems—PEDOT:PSS, WO_3_, and n‐PBDF with vertical structure as shown in Figure 1b, confirming the reliability of the fractional diffusion model in diverse insertion‐type systems.

This study establishes a robust experimental and theoretical basis for analyzing subdiffusive ion transport in MIEC systems. The insights gained herein offer general design principles for optimizing the performance of devices based on mixed conductors, particularly where ionic dynamics are rate‐limiting or memory effects are desirable.^[^ ^34^, ^35^, ^36^, ^37^ ^]^

## 2 Theory

### 2.1 Ordinary Diffusion

We recall that the ordinary diffusion model is based on two equations: the conservation equation.

(1) $$\frac{\partial n\left(x,t\right)}{\partial t}=-\frac{\partial J\left(x,t\right)}{\partial x}$$

and Fick's law for the diffusion flux

(2) $$J\left(x,t\right)=-D_{0}\frac{\partial n\left(x,t\right)}{\partial x}$$

In these equations, *n* is the diffusing carrier density ( cm^−3^), *J* is the particle flux ( cm^−2^ s^−1^), and *D*_0_ is the diffusion coefficient (cm^2^ s^−1^). The impedance spectroscopy and current transient for the measurement experiments, as shown in Figure 1a, under ordinary diffusion are well known, ^[^ ^19^, ^20^, ^21^, ^22^, ^23^, ^24^ ^]^ and the topic has been summarized.^[^ ^25^ ^]^

### 2.2 Anomalous Diffusion Model

The model of Bisquert and Compte ^[^ ^31^ ^]^ for anomalous diffusion for the electrochemical charging of a film is based on a fractional diffusion equation, which extends the ordinary conservation equation as

(3) $$\frac{\partial^{\beta }n\left(x,t\right)}{\partial t^{\beta }}=-\frac{\partial J_{\beta }\left(x,t\right)}{\partial x}$$

by application of the fractional derivative that is defined as^[^ ^38^ ^]^

(4) $$\frac{\partial^{\beta }X\left(x,t\right)}{\partial t^{\beta }}=\frac{1}{\Gamma \left(1-\beta \right)}\int_{0}^{t}{\left(t-\tau \right)}^{-\beta }\frac{\partial X\left(x,\tau \right)}{\partial \tau }d\tau$$

where the fractional exponent 0 < β < 1. The model uses the following quantities: the diffusing carrier density *n* (cm^−3^), fractional flux *J*_β_ (cm^−2^ s^−β^), and fractional diffusion coefficient *D*_β_ (cm^2^ s^−β^). The constitutive equation, on the other hand, remains in the classical form

(5) $$J_{\beta }\left(x,t\right)=-D_{\beta }\frac{\partial n\left(x,t\right)}{\partial x}$$

By combining both equations, we obtain

(6) $$\frac{\partial^{\beta }n\left(x,t\right)}{\partial t^{\beta }}=D_{\beta }\frac{\partial^{2}n\left(x,t\right)}{\partial x^{2}}$$

The electrochemical potential of the inserted species is µ  =  *qV*, where *q* is the elementary charge and *V* is the applied voltage.^[^ ^39^ ^]^ The concentration depends on the electrochemical potential by an equilibrium function $\bar{n}\left(\bar{V}\right)$. For a small perturbation of a background potential $V=\bar{V}+\Delta V$, the charge is related to the voltage as

(7) $$q\;\Delta n=c_{\mu }\Delta V$$

where *c*_µ_ (F cm^−3^) is the chemical capacitance per unit volume, which is the derivative of the concentration of a species with respect to the electrochemical potential^[^ ^40^, ^41^, ^42^ ^]^

(8) $$c_{\mu }=q{\left(\frac{d\bar{n}}{d\bar{V}}\right)}_{\bar{V}}$$

On the other hand, the electrical current density is *i*_β_(*x*,*t*)  =  *q* *J*_β_(*x*,*t*) (in A s^β − 1^ cm^−2^). Equations (3) and (4) can be converted into electrochemical quantities as follows

(9) $$c_{\mu }\frac{\partial^{\beta }\Delta V\left(x,t\right)}{\partial t^{\beta }}=-\frac{\partial \Delta i_{\beta }\left(x,t\right)}{\partial x}$$

(10) $$\Delta i_{\beta }\left(x,t\right)=-c_{\mu }D_{\beta }\frac{\partial \Delta V\left(x,t\right)}{\partial x}$$

and Equation (6) becomes

(11) $$\frac{\partial^{\beta }\Delta V\left(x,t\right)}{\partial t^{\beta }}=D_{\beta }\frac{\partial^{2}\Delta V\left(x,t\right)}{\partial x^{2}}$$

The ions are inserted at *x*  =  0, and the fractional electrical current is

(12) $$I_{\beta }\left(t\right)=i_{\beta }\left(0,t\right)=q\;J_{\beta }\left(x=0\right)$$

Their diffusion is bounded at *x*  =  *L* (in cm), the thickness of the film. Therefore, we have the second boundary condition

(13) $${\left.\frac{\partial V\left(x,t\right)}{\partial t}\right|}_{x=L}=0,t>0$$

### 2.3 Impedance

The impedance corresponds to the ratio of voltage to current in a small perturbation signal of angular frequency ω. As shown in Appendix 1, the impedance of spatially restricted diffusion is obtained by the solution of Equations ((9), (10), (11), (12), (13)). It has the form^[^ ^31^ ^]^

(14) $$Z_{d}\left(s\right)=R_{d}{\left(\frac{\omega_{d}}{s}\right)}^{\beta /2}\coth\left[{\left(\frac{s}{\omega_{d}}\right)}^{\beta /2}\right]$$

where $s=\sqrt{-1}\;\omega$ is the Laplace variable. Here, there are three parameters: β is the fractional exponent, ω_*d*_ is a characteristic diffusion frequency in rad/s

(15) $$\omega_{d}={\left(\frac{D_{\beta }}{L^{2}}\right)}^{1/\beta }$$

and *R_d_* is a diffusion resistance in Ω.

(16) $$R_{d}=\frac{1}{C_{\mu }^{*}\;\omega_{d}}$$

where $C_{\mu }^{*}=Lc_{\mu }$ is the total chemical capacitance of the film (in F/cm^2^). Hence

(17) $$\omega_{d}=\frac{1}{R_{d}\;C_{\mu }^{*}}$$

The high frequency limit of Equation (14) is

(18) $$Z_{d}\left(s\right)=R_{d}{\left(\frac{\omega_{d}}{s}\right)}^{\beta /2}$$

and the low frequency limit is

(19) $$Z\left(s\right)=\frac{1}{3}R_{d}+\frac{1}{Q_{\beta }}s^{-\beta }$$

The first term in Equation (19) is a resistance, and the second term is the impedance of a constant phase element (CPE) of parameter

(20) $$Q_{\beta }=\frac{1}{R_{d}\omega_{d}^{\beta }}$$

with unit Ω^−1^ *s* ^β^ = F *s* ^β − 1^, which becomes a pure capacitance only when β  =  1. We can write the characteristic diffusion frequency from this relation as:

(21) $$\omega_{d}=\frac{1}{{\left(R_{d}Q_{\beta }\right)}^{\beta }}$$

We can also write

(22) $$Q_{\beta }={\left(R_{d}\right)}^{\beta -1}\;{\left(C_{\mu }^{*}\right)}^{\beta }$$

This last formula enables the conversion of the measured CPE to a regular chemical capacitance via

(23) $$C_{\mu }^{*}={\left(R_{d}\right)}^{1/\beta -1}\;{\left(Q_{\beta }\right)}^{1/\beta }$$

To obtain a regular diffusion coefficient *D*_0_ we define

(24) $$\omega_{d}=\frac{D_{0}}{L^{2}}$$

Comparing with Equation (15)

(25) $$D_{0}={\left(D_{\beta }\right)}^{\beta }L^{2\left(1-\beta \right)}$$

**Figure 2:**
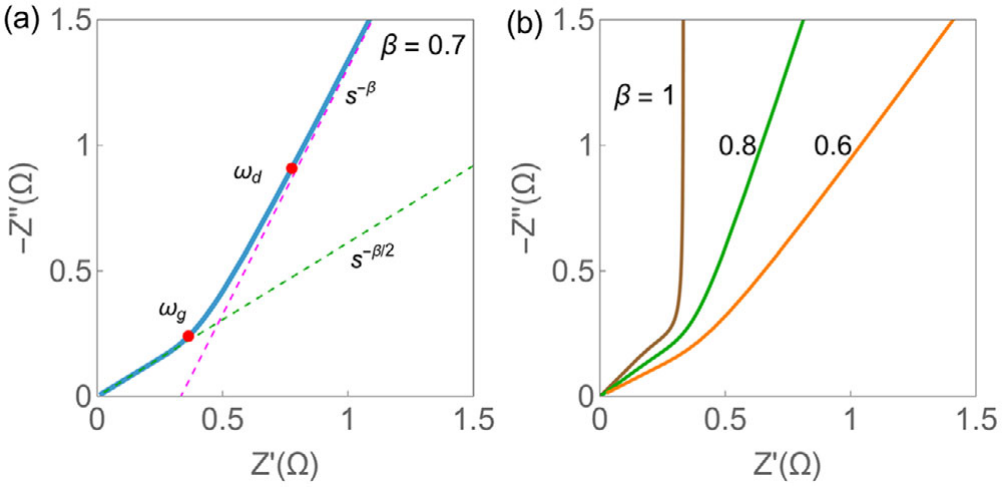
Simulation plots showing: a) Features of the impedance spectrum of the anomalous diffusion model, Equation (14), showing the characteristic frequencies and the limit functional behaviors. b) Effect of the fractional exponent on the impedance spectra.

As shown in **Figure** **2**a, the impedance is composed of two straight lines, one with exponent β/2 at high frequency, and another one with exponent β at low frequency, therefore, it obeys a property of doubling exponents.^[^ ^32^, ^43^ ^]^ Figure 2b shows that the spectra become more inclined when β decreases, while still maintaining the doubling exponent feature. Consequently, the lower β values increase the dissipation characteristic of the impedance at low frequency (since the resistive component increases). By an extension of the criterion of ref. [25], the frequency of turnover between both limit regimes is

(26) $$\omega_{g}^{\beta }=\frac{\pi^{2}}{2}\;\omega_{d}^{\beta }$$

Hence

(27) $$\omega_{g}={\left(\frac{\pi^{2}}{2}\right)}^{1/\beta }\;\omega_{d}$$

### 2.4 Transmission Line Model

**Figure 3:**
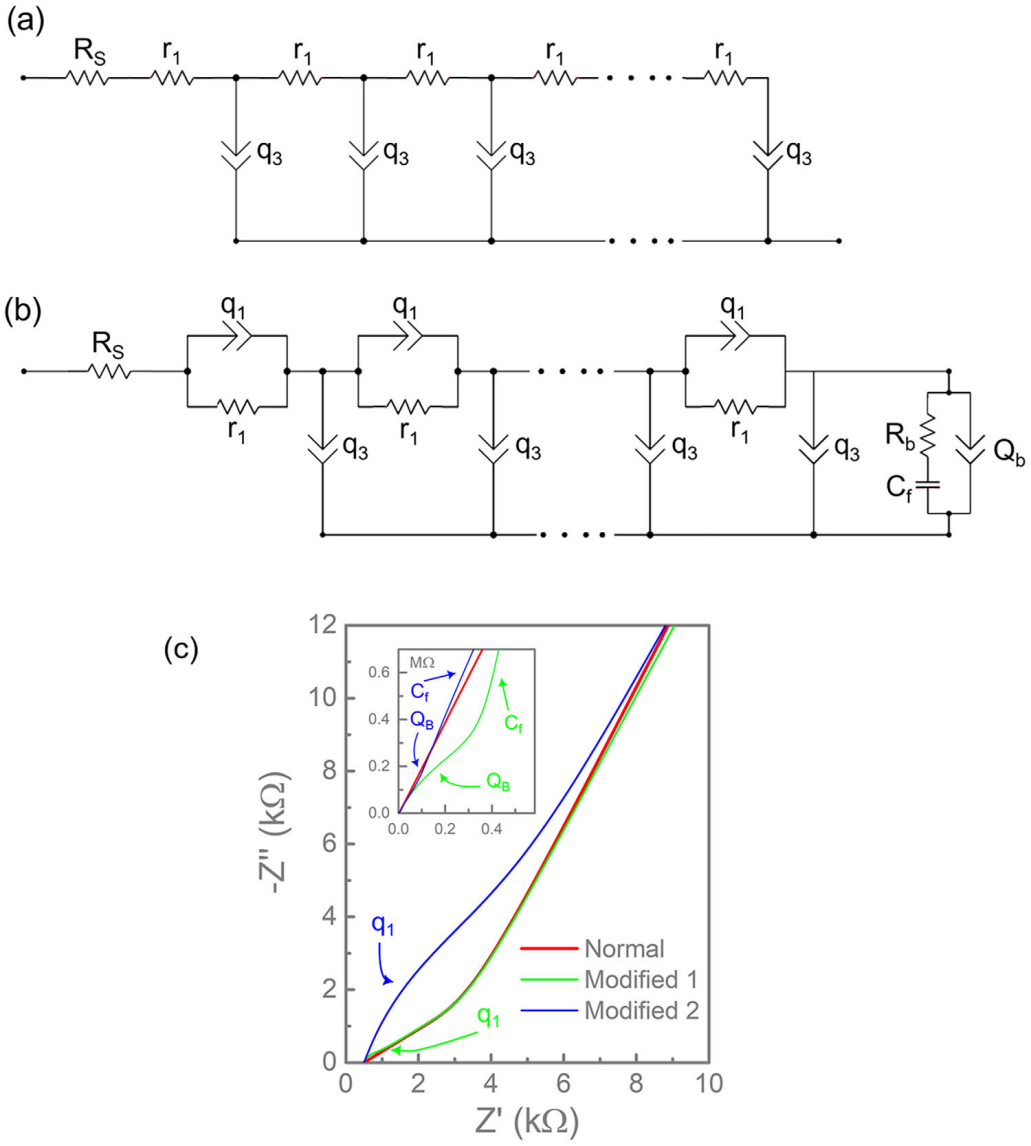
Transmission line equivalent circuit. a) Anomalous diffusion model. b) Anomalous diffusion with additional transport and boundary dynamical components. c) (c) Simulated complex plane Nyquist plots using the two different models (a, b). The “Normal” spectrum corresponds to the standard transmission line model shown in (a) while the “Modified” spectra are based on the extended transmission line model of (b). All simulations share the same set of parameters for the classical transmission line elements, i.e., *L*  =  1; *R_s_* = 0.5 *k*Ω; *R*_1_ = 7 *k*Ω; *Q*_3_ = 0.1 *mF* *s* ^β − 1^,  β  =  0.7. In the modified models, additional elements were introduced to account for boundary and dispersive transport effects: for Modified 1, the added elements and values were *Q*_1_ = 30 *nF* *s* ^α − 1^; α  =  0.9; *R_b_* = 1 *M*Ω; *Q_b_* = 1 µ*F* *s* ^γ − 1^; γ  =  0.8; *C_f_* = 1 *mF*, for Modified 2, the same elements were included, but with parameters selected to overlap spectrally with the transmission line features *Q*_1_ = 30 µ*F* *s* ^α − 1^; α  =  0.9; *R_b_* = 1 *M*Ω; *Q_b_* = 10 µ*F* *s* ^γ − 1^; γ  =  0.8; *C_f_* = 100 µ*F*.

For fitting and analysis, it is useful to express the above model as a transmission line.^[^ ^31^, ^43^, ^44^ ^]^ The local elements in Figure 3a are defined as

(28) $$r_{1}=\frac{R_{d}}{L}$$

(29) $$q_{3}=\frac{Q_{\beta }}{L}$$

This model is available in *Zview* software (“Bisquert#3″). Note that if the primary fitting parameters are *R_d_*, *Q_d_*, then the diffusion frequency is obtained by Equation (21), and the generalized Warburg impedance in Equation (18) is

(30) $$Z\left(s\right)={\left(\frac{R_{d}}{Q_{\beta }}\right)}^{1/2}s^{-\beta /2}$$

The series resistance *R_s_* is usually a significant parameter for transient charging. The total impedance is

(31) $$Z\left(s\right)=R_{s}+Z_{d}\left(s\right)$$

The model of **Figure** **3**b contains additional features for the boundary condition and for dispersive transport.^[^ ^44^, ^45^, ^46^ ^]^ The boundary condition is associated with excess carrier injection at very low frequencies (i.e., long timescales), where real capacitive contributions from the substrate (denoted *C_f_*) become comparable to, or even dominate over, the injected carrier dynamics. These effects also include the dispersive capacitance arising from charge accumulation at the MIEC/substrate interface, typically represented as a distributed double layer with elements *R_b_Q_b_*. On the other hand, dispersive transport phenomena are captured in the model by introducing an additional CPE *Q*_1_ within the transmission line itself. This element accounts for a frequency‐dependent conductivity.^[^ ^47^ ^]^ The more general model in Figure 3b has been found necessary to correctly fit the impedance data. In Figure 3c, the standard transmission line model (in Figure 3a) is compared to two modified situations, as shown (Figure 3b). In Modified 1, the parameters are chosen such that the boundary and dispersive transport elements are spectrally decoupled from the transmission line, while in Modified 2, they overlap, demonstrating how these additional elements reshape the impedance spectrum. These features appear to be quite frequent in the intercalation films, as we discuss in the Results section.

### 2.5 Transient Current in Response to a Voltage Step

We search for the solution of the current under a voltage step Δ*V* = *V*_0_ with zero initial conditions

(32) $$\Delta V\left(x,0\right)=0,0<x<L$$

and Equation (13). The current density (in A cm^−2^) is given by the expression

(33) $$I_{d}\left(t\right)=-\omega_{d}^{1-\beta }D_{\beta }c_{\mu }\frac{\partial \Delta V\left(x,t\right)}{\partial x}$$

This is the charging current until a new equilibrium situation is achieved. The result obtained in Appendix 2 is

(34) $$I_{d}\left(t\right)=\frac{2V_{0}}{R_{d}}\sum_{k=1}^{\infty }E_{\beta }\left[-\alpha_{k}^{2}{\left(\omega_{d}\;t\right)}^{\beta }\right]$$

where *E*_β_(− *z*) denotes the one‐parameter Mittag‐Leffler function^[^ ^48^ ^]^ and α_*k*_ = π(*k* − 1/2) with k=1,2,… For short times we calculate the inverse Laplace transform of *V*_0_/*sZ_d_* by considering the high‐frequency limit of the impedance function (Equation (18)).

(35) $$I_{d}\left(t\right)=\frac{V_{0}}{R_{d}\omega_{d}^{\beta /2}\Gamma \left(1-\frac{\beta }{2}\right)}t^{-\beta /2},\;t\ll \frac{1}{\omega_{d}}$$

For long times leading term in the sum in Equation (34) gives the approximation

(36) $$I_{d}\left(t\right)\;∼\;\frac{2V_{0}}{R_{d}}E_{\beta }\left[-\frac{\pi^{2}}{4}{\left(\omega_{d}t\right)}^{\beta }\right],\;t\gg \frac{1}{\omega_{d}}$$

The Mittag‐Leffler function exhibits the limiting behavior of^[^ ^49^, ^50^, ^51^ ^]^

(37) $$E_{\beta }\left(-z\right)=\sum_{k=1}^{\infty }{\left(-1\right)}^{k-1}\frac{{\left(-z\right)}^{-k}}{\Gamma \left(1-\beta k\right)},\;\beta >0,\left|z\right|\to \infty$$

and then, with the first‐order approach of *E*_β_,^[^ ^52^ ^]^ we finally obtain

(38) $$I_{d}\left(t\right)=\frac{8\;V_{0}}{R_{d}\;\omega_{d}^{\beta }\;\pi^{2}\Gamma \left(1-\beta \right)}t^{-\beta },\;t\gg \frac{1}{\omega_{d}}$$

Equation (38) gives rise to a characteristic hallmark of fractional calculus consisting of a terminal inverse power‐law decay,^[^ ^53^ ^]^ clearly linked to the Curie‐von Schweidler law.^[^ ^54^ ^]^ The full expression *I_d_* (*t*) in Equation (34) extrapolates between two inverse power‐law decays, with the previously mentioned equivalent property of doubling exponents in impedance, but, for the first time, presented here in the time domain.

**Figure 4:**
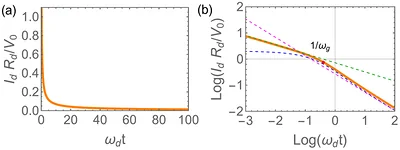
a) Current decay function, Equation (34), for β  =  0.7 b) Approximating functions in Log‐Log scale. The green line shown is the short‐time limit power law, the purple line is the long‐time power law, and the blue line is the *E*_β_[−π^2^(ω_*d*_ *t*)^β^/4] behavior. The point is the indicated characteristic time.

In **Figure** **4**, we plot the solution of the transient current in Equation (34). We also show the two separated power‐law regimes at long and short times given by Equations ((35), (37), (38)). We remark that 1/ω_*g*_ is the characteristic time separating both domains. It is obvious that in the limit β  =  1, Equation (35) reduces to the Cottrell‐type dynamic response, $I_{d}\left(t\right)=V_{0}/R_{d}\sqrt{\pi \omega_{d}t}$. Consideration of the series resistance effects on the potentiostatic transients modifies the boundary condition given by Equation (12), resulting in

(39) $$V\left(0,t\right)=V_{0}-I\left(0,t\right)R_{s},\;t>0$$

## 3 Experimental Section

### 3.1 Materials and Device Structures

The vertical conductor‐electrolyte device structure was schematically shown in Figure 1b. Three types of active materials—PEDOT:PSS, WO_3_, and n‐doped PBDF—were investigated. Gold (Au) was used as the bottom electrode for PEDOT:PSS devices due to its high work function and excellent ohmic contact with p‐type polymers. For WO_3_ and n‐PBDF films, indium tin oxide (ITO) substrates were employed to ensure transparent, low‐resistance contact suitable for n‐type charge transport.

### 3.2 Preparation of PEDOT:PSS Dispersion and Device Fabrication

#### 3.2.1 PEDOT:PSS Dispersion Preparation

Clevios PH 1000 (Ossila Ltd.) was mixed with 6% v/v ethylene glycol (EG) and 1% v/v (3‐glycidyloxypropyl)trimethoxysilane (GOPS) (Sigma–Aldrich). EG improves electronic conductivity, while GOPS acts as a chemical cross‐linker, enhancing aqueous stability. The mixture was sonicated for 10 min and filtered through a 0.45 µm hydrophilic PTFE membrane.^[^ ^16^ ^]^ Cross‐linking creates covalent bonds between polymer chains and reduces film swelling, thereby stabilizing the film‐electrolyte interface and preserving ionic pathways essential for reliable transient measurements and impedance analysis.^[^ ^55^ ^]^

#### 3.2.2 Substrate Preparation and Film Deposition

Pre‐oxidized Si/SiO_2_ wafers (300 nm SiO_2_, 15 mm  × 20 mm, Ossila Ltd.) were cleaned with acetone and IPA via ultrasonication for 15 min each. A 120 nm Au bottom electrode was deposited by chemical vapor deposition (CVD). The substrates underwent 15 min UV‐ozone (UVO) treatment before PEDOT:PSS deposition via spin‐coating at varying speeds, yielding films of five different thicknesses: 630, 200, 120, and 90 nm, named as P630, P200, P120, and P090, respectively. Films were annealed at 120 °C for 20 min to remove residual solvents and to activate the GOPS cross‐linking reaction, ensuring chemical stability during measurement.

#### 3.2.3 Device Assembly

A 3D‐printed adhesive well (7 mm × 3 mm inner diameter, effective area: 21 mm^2^) was mounted on each sample to confine the electrolyte. 300 µL of 0.1 m NaCl aqueous solution was added as the electrolyte, with an Ag/AgCl electrode as the top contact. The electrolyte well configuration in a vertical two‐terminal assembly was presented in Figure S5 (Supporting Information).

### 3.3 Preparation of WO_3_ Dispersion and Device Fabrication

#### 3.3.1 WO_3_ Dispersion Preparation

WO_3_ nanopowder (90–100 nm, Ossila) was dispersed in a solvent mixture (600 µL IPA and 300 µL DI water) along with 100 µL of 3 wt.% polyvinyl alcohol (PVA, Mw ≈88000–97000, Sigma–Aldrich) as binder. The suspension (containing 100 mg WO_3_) was sonicated for 30 min to ensure homogeneity.

#### 3.3.2 Film Deposition

ITO‐coated glass substrates (15 mm × 20 mm) were cleaned and UVO‐treated for 15 min. Spin coating was performed at 1000 and 3000 rpm for 40 s, respectively. After deposition, the films were dried at 70 °C for 15 min, followed by annealing at 300 °C for 1 h to remove the PVA binder and form porous, binder‐free WO_3_ layers. The final film thicknesses were 4.7 and 3.5 µm, as listed in Table S1 (Supporting Information).

#### 3.3.3 Device Configuration

Vertical Structure: Similar to the PEDOT:PSS device, a 3D‐printed adhesive well with an inner diameter of 7mm*3mm was attached to confine the electrolyte. A 0.1 m H_2_SO_4_ aqueous solution was used as the electrolyte (300 µL), with an Ag/AgCl electrode serving as the top electrode.

Three‐electrode measurement: The three‐electrode configuration uses a coiled platinum (Pt) wire as the counter electrode (CE) and an Ag/AgCl electrode as the reference (RE). WO_3_‐ITO was connected as the working electrode (WE). Three electrodes were immersed in 0.1 m H_2_SO_4_ aqueous electrolyte.

### 3.4 Fabrication of n‐Doped PBDF Film and Device

#### 3.4.1 n‐PBDF Polymer Synthesis

The n‐PBDF polymer (n‐doped benzo[1,2‐b:4,5‐b']difuran‐2,6‐dione polymer) was synthesized via TMQ‐catalyzed oxidative polymerization of BDF monomer under N_2_ atmosphere, followed by in situ water‐mediated doping.^[^ ^56^ ^]^ The resulting black polymer ink (15 mg mL^−1^ in DMSO) was purified by dialysis (cutoff 10 kDa) and stored in anhydrous DMSO.

#### 3.4.2 Film Preparation

The polymer ink was sonicated and stirred at 45 °C for 1 h, then filtered through a 0.45 µm PVDF syringe filter. Cleaned ITO/glass substrates (20 mm × 15 mm) were treated with UVO (15 min). 30 µL of the filtered ink was spin‐coated at 4000 rpm for 1 min in air. The films were dried in a vacuum oven at room temperature for ≥5 h.

#### 3.4.3 Device Assembly

A polytetrafluoroethylene (PTFE) well with an inner diameter of 1 cm (active area: 0.785 cm^2^) was affixed on top of the film. An aqueous 0.1 m NaCl solution was used as the electrolyte with an Ag/AgCl electrode inserted into the well serving as the top contact. The electrical conductivity of the as‐prepared n‐PBDF was measured by the 4‐point probe method in a thin film, giving a value of 170 S cm^−1^.

### 3.5 Electrical and Morphological Characterization

#### 3.5.1 Electrochemical Measurements

Impedance spectroscopy (IS) and chronoamperometry (CA) were performed using a Metrohm Autolab PGSTAT204 potentiostat equipped with an FRA32M module (controlled via Nova 2.1 software). IS was carried out in the 10^5^ − 0.1 Hz range (1 Hz cutoff for WO_3_ vertical devices) with 10 mV AC perturbation. The full Bode impedance spectra (magnitude |Z| and phase angle vs log frequency) are provided in (Figures S2, S6, and S10, Supporting Information). CA was performed after a small potential step.

The polarity of the applied bias was selected to match the direction of ionic injection known to occur in each material system. For PEDOT:PSS and n‐doped PBDF, small positive biases promote cation migration from the electrolyte into the film, enabling volumetric ionic response.^[^ ^11^ ^]^ In contrast, WO_3_ exhibits strong ionic uptake under negative bias, which was a well‐established feature of electrochromic oxides.^[^ ^57^, ^58^ ^]^ All applied potentials were within the stable electrochemical window and produced reproducible ion‐injection transients suitable for fractional diffusion analysis.

#### 3.5.2 Thickness and Morphology Analysis

PEDOT:PSS: Thickness and surface morphology (see Figure S4, Supporting Information) were measured by contact‐mode AFM (Bruker Multimode 8 with NanoScope V controller). Thickness was tuned by spin speed and confirmed by AFM (see Table S3, Supporting Information).

WO_3_: Surface morphology (see Figure S8, Supporting Information) was imaged using FESEM (ZEISS ULTRA 55).

n‐PBDF: Film thickness was measured using a profilometer.

## 4 Results and Discussion

### 4.1 Anomalous Diffusion Behavior in MIEC‐Electrolyte System

To establish the general validity of the fractional diffusion methodology, we applied identical impedance spectroscopy (IS) and chronoamperometry (CA) measurements to three representative MIEC systems: PEDOT:PSS, WO_3_, and n‐doped PBDF films, all configured in vertically confined geometries. All three systems exhibit consistent signatures of anomalous diffusion under electrochemical charging, as revealed by both impedance spectroscopy and transient current measurements.

**Figure 5:**
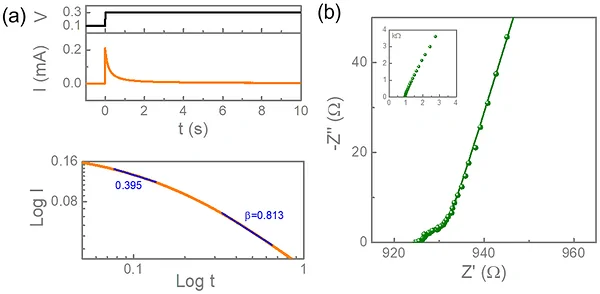
Transient current response and IS of the PEDOT: PSS film‐electrolyte vertical system with sample P200. a) Transient current response and its logarithmic plot curve (with linear slopes in blue) obtained through CA measurements. The applied potential is set for a step voltage initially *V*_I_ = 0.1 *V* and Δ*V*  =  0.2 *V*, as indicated in the black line. b) Nyquist plot of the impedance data of the system under applied potential 0.3 *V*. The green circles represent the experimental result, while the line corresponds to the fitted curve using the DX Type 12 – Bisquert model. The inset figure shows the full spectrum.

Transient measurements performed on PEDOT:PSS films following a small voltage step reveal distinct non‐exponential current decay profiles governed by ionic diffusion. As illustrated in **Figure** **5**a, the current response upon a small potential step (*V*_I_ = 0.1 *V*, Δ*V*  =  0.2 *V*) exhibits rapid initial decline, followed by a slower decay. The log‐log plot in Figure 5a displays two linear regimes, corresponding to short‐ and long‐time dynamics, with fitted slopes ‐0.395 and ‐0.813, respectively. From the late‐time decay region, we extract the fractional exponent as β  =  0.813. These values are in strong agreement with the theoretical predictions derived from the fractional diffusion model shown in Equations ((34), (35), (36), (37), (38)), where both short and long‐time responses adhere to a power‐law decay. The two components demonstrate the characteristic “exponent doubling” (β and β/2).

Simultaneously, IS measurements in Figure 5c provide complementary insights. The  complex plane impedance plot displays a transition from frequency power‐laws at high‐frequency and low frequency, as predicted in Figure 2a. The fitted transmission line using the DX Type 12 – Bisquert#3 model based on anomalous diffusion impedance (Equation 14, Figure 3a) yields an exponent β  =  0.811, consistent with the long‐time transient analysis. The extracted parameters are summarized in **Table** **1** and Table S1 (Supporting Information). Furthermore, the values of the chemical capacitance of 100–200 F cm^−3^ shown in Table S2 (Supporting Information) are in agreement with the reports in the literature.^[^ ^59^, ^60^, ^61^ ^]^

**Table 1:** Device characteristics and diffusion frequencies.

Device	*L* (nm)	β *impedance*	β *transient*	ω_*d*_ (*rad*/*s*) *impedance*	ω_*d*_ (*rad*/*s*) *transient*
P630	630	0.801	0.793	15.5	1.33
P200	200	0.811	0.813	63.2	4.84
P120	120	0.849	0.807	90.1	7.29
P090	90	0.904	0.853	190	11.8
WO_3_ a	4740	0.817	0.866	43.9	25.6
WO_3_ b	3540	0.819	0.826	24.1	18.8
n‐PBDF	21	0.717	0.721	3.33	57.4

The two fractional exponents β of transient current and impedance are approximately equal (0.813 and 0.811), revealing that they are strongly correlated, confirming a close agreement between CA and IS that validates the theoretical correspondence between time and frequency domains for anomalous diffusion processes, which has been presented here in Sec. 2.

**Figure 6:**
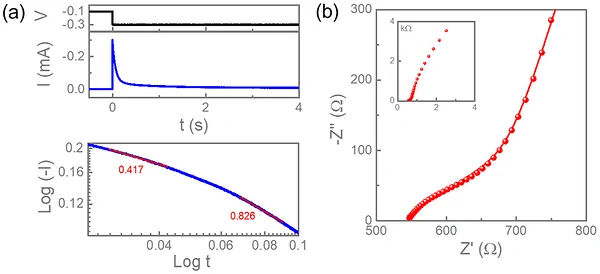
Transient current response and IS of the WO_3_ film‐electrolyte vertical system. a). WO_3_ transient current response and its logarithmic plot curve (with linear slopes in red) obtained through CA measurements. The applied potential is set for a step voltage initially *V*_I_ = −0.1 *V* and Δ*V*  =   − 0.2 *V*, as indicated in the black line. b) Nyquist plot of the impedance data of WO_3_ under applied potential − 0.3 *V*. The red circles represent the experimental result, while the line corresponds to the fitted curve using the DX Type 12 – Bisquert model. The inset figure shows the full spectrum. Frequency ranges from 10^5^ Hz to 1 Hz. The data for another thickness system is shown in Figure S6 (Supporting Information).

**Figure** **6** presents the impedance spectra and transient decay of WO_3_ films in a two‐terminal structure. The transient current follows a power‐law decay with slopes corresponding to −0.417 at short times and −0.826 at long times, yielding a long‐time exponent β  =  0.826 as shown in Figure 6a. The complex plane plots exhibit a typical low‐frequency slope characteristic of anomalous diffusion. Fitting the data with the fractional transmission‐line model yielded a β value of 0.819, closely matching the transient decay exponent of 0.826, where log‐log current‐time plots exhibited two characteristic power‐law regimes. This agreement confirms that both frequency‐ and time‐domain data reflect the same ionic transport process. In addition, WO_3_ measured in a standard three‐electrode configuration (Figures S8 and S9, Supporting Information) also displayed clear fractional transient behavior, further supporting the generality of the observed anomalous dynamics.

**Figure 7:**
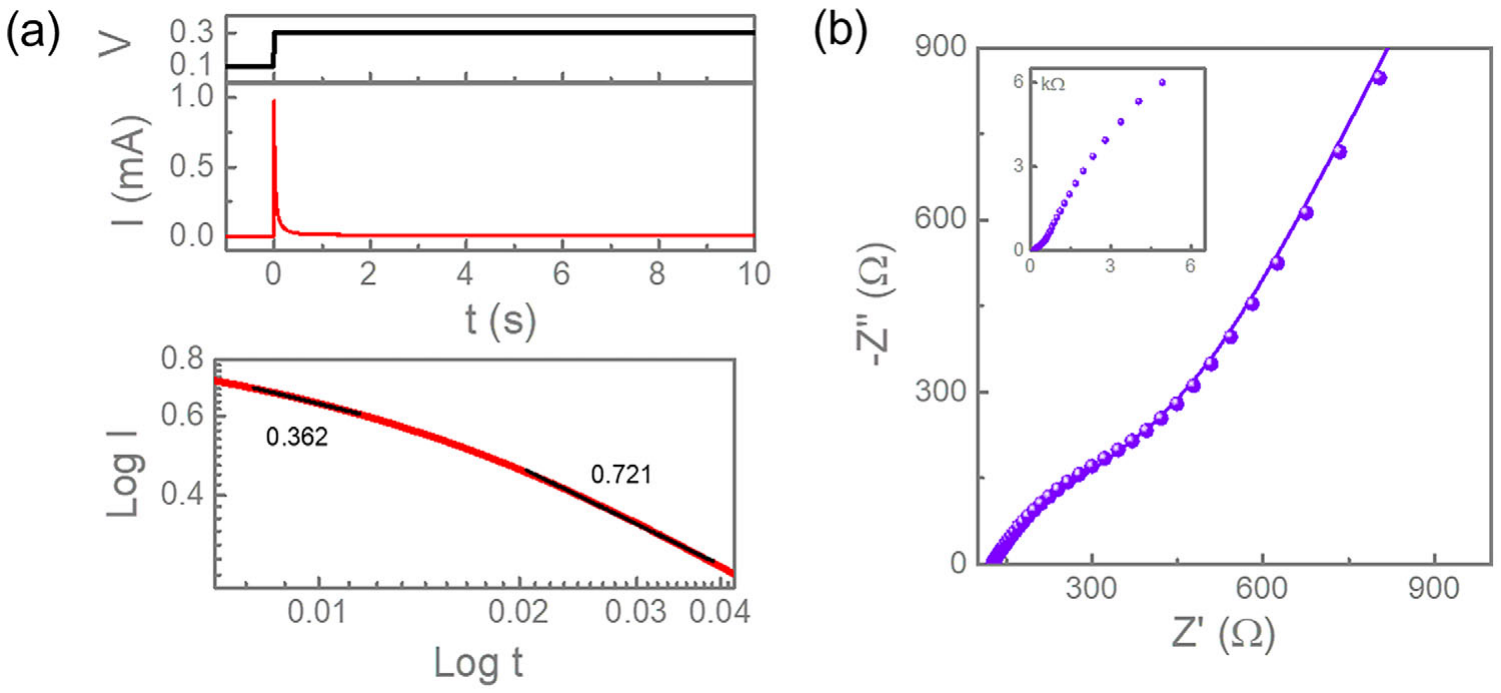
Transient current response and IS of the n‐PBDF film‐electrolyte vertical system. a). Transient current response and its logarithmic plot curve (with linear slopes in black) obtained through CA measurements. The applied potential is set for a step voltage at initially *V*_I_ = 0.1 *V* and Δ*V*  =  0.2 *V*, as indicated in the black line. b) Nyquist plot of the impedance data under applied potential 0.3 *V*. The purple circles represent the experimental result, while the line corresponds to the fitted curve using the DX Type 12 – Bisquert model. The inset figure shows the full spectrum.

Similarly, **Figure** **7** presents results for n‐type PBDF films. The impedance data (Figure 7b) reveal fractional diffusion behavior with an extracted exponent of β  =  0.717. The corresponding transient current decay (Figure 7a) gives a nearly identical β  =  0.721. This strong agreement further confirms the internal consistency of the methodology in a high‐performance n‐type polymer system.

Consistent β values extracted from both IS and CA measurements were observed across all three material systems‐PEDOT:PSS, WO_3_, and n‐PBDF‐despite their distinct compositions and morphologies. This highlights the versatility of the fractional diffusion model as a general framework for analyzing anomalous ionic transport in MIEC films. In the following section, we further explore how film thickness influences the diffusion response, using PEDOT:PSS as a representative system.

### 4.2 Impedance Data Fitting and Response Across Varying PEDOT:PSS Film Thicknesses

To generalise the observed anomalous behavior, we conducted a systematic analysis across PEDOT:PSS films of varying thickness: P090, P120, P200, and P630 (corresponding to *L*≅ 90–630 nm). All films exhibited the frequency‐domain and time‐domain features of anomalous diffusion.

**Figure 8:**
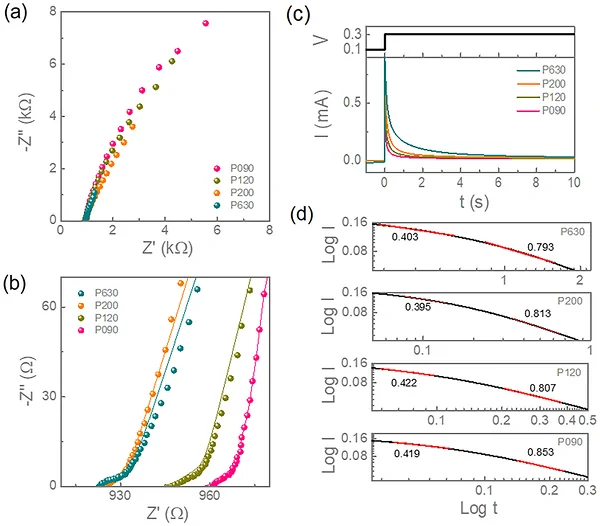
a) Nyquist plot of the impedance data of different thickness samples under applied potential 0.3 *V* with frequency range from 10^5^ Hz to 0.1 Hz, b) High frequency range of the impedance data showing the transmission line part. The circles represent the experimental result, while the line corresponds to the fitted curve using the DX Type 12 – Bisquert#3 model. c) Transient current response of the PEDOT: PSS film‐electrolyte vertical system across different samples. d) Logarithmic plot curve (with linear slopes in red) of the transient current response under a step voltage at initially *V*_I_ = 0.1 *V* and Δ*V*  =  0.2 *V* as indicated. (Another series of data under potential 0.5 *V* can be seen in Figure S1, Supporting Information).

**Figure** **8** presents representative complex plane impedance plots for all devices, each exhibiting the characteristic features of fractional diffusion. The fitted parameters from the DX Type 12 – Bisquert∖#3 model (Figure 3b) confirm the presence of transmission‐line‐like behavior with fractional‐order impedance. The list of extracted parameters is presented in Table 1 and Table S1 (Supporting Information).

As in Figure 8a, thicker samples show a semi‐circular trend at low frequencies, indicative of higher ionic resistance, while the high‐frequency regime (Figure 8b) consistently reveals a transmission line pattern. The extracted exponents from IS measurements remain in the range 0.801 ≤ β ≤ 0.904 (see Table 1), confirming the persistence of anomalous diffusive transport across varied geometries. This range is also consistent with the exponents obtained from transient current decay. The doubling of exponents in both domains constitutes a robust experimental signature for fractional ion dynamics in MIEC‐electrolyte systems.

Transient decay in Figure 8c demonstrates faster current decay in thinner films, with a clear shift in characteristic time to shorter values. In Figure 8d, a progressive shift of the system's diffusive response toward higher frequencies and shorter characteristic timescales is observed. This corresponds to a faster ionic equilibration process in thinner films, where the reduced diffusion path and minimized volumetric resistance facilitate more rapid charge redistribution. This trend is consistent with expectations for confined vertical geometries, where film thinning directly enhances ionic transit time, Eq. (15).

To quantitatively assess the consistency between time‐ and frequency‐domain analyses, Table 1 summarizes the extracted parameters for all measured devices. The fractional exponent β obtained from impedance spectroscopy and transient current decay shows strong agreement across the PEDOT:PSS series and extends to chemically and structurally distinct systems, including WO_3_ and n‐doped PBDF. The discrepancy between β values is typically below 0.05, confirming the robustness of the fractional analysis. Similarly, the ω_*d*_ derived from both techniques display the same trend with respect to film thickness and material system, despite absolute differences in value. These consistent results confirm that both IS and CA probe the same underlying transport mechanism and validate the general applicability of the fractional diffusion model to various insertion‐type MIECs.

**Figure 9:**
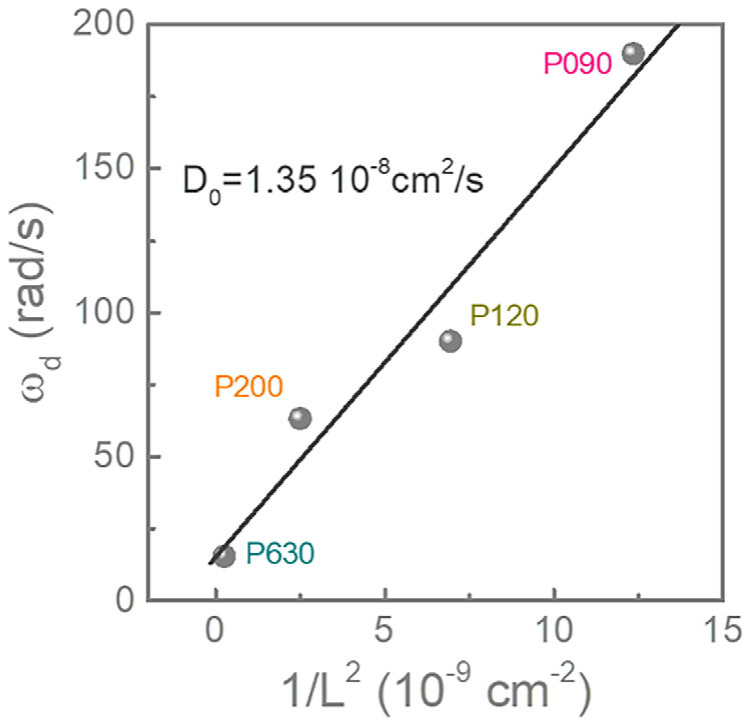
The plot for ω_*d*_ extracted from IS fitting as a function of 1/*L* ^2^ with different‐thickness samples. The linear fit supports a diffusion‐controlled process. The slope corresponds to the diffusion coefficient (*D*_β_) as indicated.

As listed in Table S1 (Supporting Information), the generalized diffusion coefficient (*D*_β_) ranged in 6.59  × 10^−9^ cm^2^ *s* ^−β^ to 3.55  × 10^−8^ cm^2^ *s* ^−β^, where thicker films alleviate confinement effects, allowing more rapid transit time. The complete dataset across all PEDOT:PSS thicknesses (P090–P630) demonstrates internally consistent β exponents and diffusion coefficients, reinforcing the robustness of the fractional diffusion framework. **Figure** **9** displays the ω_*d*_ plotted as a function of 1/*L* ^2^, revealing a linear relationship. This supports the scaling law predicted by Equation (24), indicating that ionic transport in all samples is predominantly governed by a thickness‐limited diffusion process. The slope of this fit corresponds to the effective regular coefficient *D*_0_ = 1.35  × 10^−8^ cm^2^ s^−1^, further confirming the physical meaning of ω_*d*_ as a geometry‐compensated dynamic descriptor.

## 5 Discussion

The analysis confirms that ionic transport in these films follows a thickness‐limited anomalous diffusion process, which can be reliably captured by the fractional diffusion model. Moreover, the fractional impedance response offers a practical diagnostic for identifying diffusive behavior and optimising operational regimes. These insights bridge theoretical electrochemistry and practical device engineering, illustrating how transport dimensionality can be engineered by tuning film thickness and morphology.

A closer inspection of the impedance spectra in Figures 5, 6, 7, 8 and Supporting Information reveals that the theoretical model in Figures 2 and 3a, derived from the fractional diffusion equation, does not fully capture the entire frequency range of the experimental data. At high frequencies, an additional arc is typically observed due to dispersive conduction effects (*Q*_1_), while low‐frequency curvature often arises from boundary phenomena or interfacial capacitance, as discussed in Sec. 2.4. These elements, although external to the fractional diffusion process, can overlap with the diffusion regime and shift the apparent ω_*d*_ extracted from charging transients. As summarized in Table 1 and illustrated in Figure S3 (Supporting Information), the ω_*d*_ values obtained from IS and CA exhibit systematic shifts but preserve their material‐ and geometry‐dependent trends. This divergence in nominal values reflects differing dominant capacitive contributions in each technique, yet both consistently reproduce the anomalous decay behavior defined by β. For more detailed analysis of these secondary contributions and their effects on the impedance response, refer to the Supporting Information.

Taking all of this into account, we establish a robust connection between the anomalous diffusive behavior observed in impedance spectroscopy and that extracted from transient charge measurements. In this way, in the systems measured in this work, it has been possible to reliably capture the diffusion process, as shown in the thickness dependence of the characteristic frequency obtained in Figure 9.

Although our analysis is performed in a simplified vertical structure film‐electrolyte system, the consistent agreement between impedance and transient responses across three chemically and structurally distinct MIEC systems—PEDOT:PSS, WO_3_, and n‐PBDF—demonstrates the robustness and generality of the fractional diffusion framework. Importantly, this consistency is preserved despite differences in electrode type, polarity, and dimensional confinement, confirming that essential features of anomalous ion transport can still be reliably extracted under constrained conditions.

While the findings are not intended to replicate full device‐level behavior—as would be the case in OECTs, where lateral transport, gating effects, and multi‐interface coupling play critical roles—they nonetheless establish a foundational framework for interpreting diffusion‐limited kinetics. The ability to extract physically meaningful parameters from either frequency‐ or time‐domain data opens new opportunities for both fundamental studies and device optimization, particularly in the context of emerging electrochemical and bioelectronic applications. This dual‐method approach represents a significant advance in methodology and underscores the value of integrating different characterization tools to uncover universal transport features in complex MIEC systems.

## 6 Conclusion 

This work presents a unified approach to analyze anomalous ionic transport in mixed ionic‐electronic conductors (MIECs) by combining impedance spectroscopy and transient current measurements through a fractional diffusion model. By applying this approach to three representative systems—PEDOT:PSS, WO_3_, and n‐PBDF—we demonstrated consistent power‐law responses in both time and frequency domains, confirming the generality of anomalous diffusion.

Strong agreement between exponents β derived from impedance spectroscopy and chronoamperometry supports the physical basis of the fractional model. This consistency supports the reliability of the model and shows its usefulness for extracting meaningful diffusion parameters.

Our approach bridges electrochemical theory and practical experimentation, offering a reliable and reproducible method to quantify anomalous diffusion charging dynamics in MIEC‐based devices. While the experimental configuration captures key features relevant to vertical structure systems—such as volumetric ion penetration, mixed conduction, and time/frequency response—it remains a simplified model with certain geometric and biasing constraints. This work lays a foundation for future studies on tuning ionic‐electronic coupling via structural control and motivates the integration of fractional models in device simulation and the design of next‐generation energy and electronic devices.

## Appendix 1

### Fractional Impedance Function

In the derivation, we remove the variation Δ sign as we are focused only on small perturbation quantities. We have *n*  =  Δ*n*,  *V*  =  Δ*V*, etc. Starting from

(40) $$\frac{\partial^{\beta }n\left(x,t\right)}{\partial t^{\beta }}=D_{\beta }\frac{\partial^{2}n\left(x,t\right)}{\partial x^{2}}$$

we take the Laplace transform, which gives

(41) $$s^{\beta }n=D_{\beta }\frac{\partial^{2}n}{\partial x^{2}}$$

with the assumption of zero initial conditions. This can be written as

(42) $$\frac{d^{2}n}{dx^{2}}=\frac{1}{\lambda^{2}}n$$

where

(43) $$\lambda ={\left(\frac{D_{\beta }}{s^{\beta }}\right)}^{1/2}$$

The general solution is given by

(44) $$n=n_{1}\cosh\left(\frac{x}{\lambda }\right)+n_{2}\sinh\left(\frac{x}{\lambda }\right)$$

Applying the boundary conditions, we obtain

(45) $$I_{\beta }=-\frac{q\;D_{\beta }\;n_{2}}{\lambda }$$

and

(46) $$n_{1}=-n_{2}\;\mathrm{cotanh}\left(\frac{L}{\lambda }\right)$$

The impedance is given by the expression

(47) $$Z_{\beta }=\frac{V}{I_{\beta }}$$

The voltage is

(48) $$V\left(0\right)=\frac{q}{c_{\mu }}n_{1}$$

and the impedance is thus

(49) $$Z_{\beta }=\frac{\lambda }{c_{\mu }D_{\beta }}\coth\left(\frac{L}{\lambda }\right)$$

where again

(50) $$\lambda ={\left(\frac{D_{\beta }}{s^{\beta }}\right)}^{1/2}$$

Now we can introduce a characteristic frequency

(51) $$\omega_{d}={\left(\frac{D_{\beta }}{L^{2}}\right)}^{1/\beta }$$

in rad/s, that corresponds to the inverse of the transit time of the diffusing particles.^[^ ^25^ ^]^ We thus have

(52) $$\lambda ={\left(\frac{\omega_{d}}{s}\right)}^{\beta /2}L$$

Equation (49) becomes

(53) $$Z_{\beta }=\frac{L}{c_{\mu }D_{\beta }}{\left(\frac{\omega_{d}}{s}\right)}^{\beta /2}\coth\left[{\left(\frac{s}{\omega_{d}}\right)}^{\beta /2}\right]$$

Introducing the resistance

(54) $$R_{\beta }=\frac{L}{c_{\mu }D_{\beta }}$$

(in units Ω s^β ‐1^ cm^2^) one can now express Equation (53) in the standard form^[^ ^31^ ^]^

(55) $$Z_{\beta }=R_{\beta }{\left(\frac{\omega_{d}}{s}\right)}^{\beta /2}\coth\left[{\left(\frac{s}{\omega_{d}}\right)}^{\beta /2}\right]$$

The impedance has the units of *R*_β_. We also note that

(56) $$R_{\beta }=\frac{1}{C_{\mu }^{*}\omega_{d}^{\beta }}$$

where $C_{\mu }^{*}=L\;c_{\mu }$ is the total chemical capacitance of the film (in F/cm^2^). Therefore, the characteristic frequency can be written

(57) $$\omega_{d}=\frac{1}{{\left(R_{\beta }\;C_{\mu }^{*}\right)}^{1/\beta }}$$

### Regular Impedance Function

To obtain a regular diffusion impedance function *Z_d_* (in Ω cm^2^) that corresponds to the output of the impedance meter, we introduce a current in A cm^−2^:

(58) $$I_{d}=\omega_{d}^{1-\beta }I_{\beta }$$

Then we have

(59) $$Z_{d}=\frac{V}{I_{d}}=\frac{1}{\omega_{d}^{1-\beta }}Z_{\beta }$$

which takes the form

(60) $$Z_{d}=R_{d}{\left(\frac{\omega_{d}}{s}\right)}^{\beta /2}\coth\left[{\left(\frac{s}{\omega_{d}}\right)}^{\beta /2}\right]$$

where

(61) $$R_{d}=\frac{R_{\beta }}{\omega_{d}^{1-\beta }}=\frac{1}{C_{\mu }^{*}\;\omega_{d}}$$

Now *R_d_* is in Ω cm^2^, as required. The frequency

(62) $$\omega_{d}=\frac{1}{R_{d}\;C_{\mu }^{*}}$$

corresponds to the *RC* time constant of the diffusion process.

### Limit Forms of the Impedance Function

At high frequency, we have

(63) $$Z_{d}=R_{d}{\left(\frac{\omega_{d}}{s}\right)}^{\beta /2}$$

It becomes the Warburg impedance when β  =  1. At low frequency

(64) $$Z_{d}=\frac{1}{3}R_{d}+\frac{1}{Q_{\beta }s^{\beta }}$$

where the *Q*_β_ is the prefactor of a CPE impedance

(65) $$Q_{\beta }=\frac{1}{R_{d}\omega_{d}^{\beta }}$$

with unit Ω^−1^ *s* ^β^ = *F* *s* ^β − 1^. The term *Q_d_* becomes a pure capacitance when β  =  1. We also have

(66) $$Q_{\beta }={\left(R_{d}\right)}^{\beta -1}\;{\left(C_{\mu }^{*}\right)}^{\beta }$$

### Transmission Line Representation

The transmission line equations are^[^ ^31^ ^]^

(67) $$\frac{\partial \phi_{m}\left(x,t\right)}{\partial x}=-\chi_{m}i_{m}$$

(68) $$\frac{\partial i_{m}\left(x,t\right)}{\partial x}=-\frac{1}{\zeta_{m}}\phi_{m}$$

**Figure 10:**
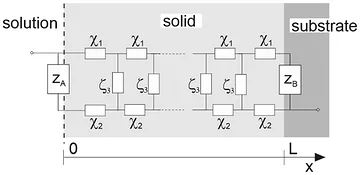
General transmission line.

These equations correspond to **Figure** **10** with χ_1_ = χ_*m*_, χ_2_ = 0,  ζ_3_ = ζ_*m*_ and the boundary conditions *Z_A_* =  *Z_B_* = 0.

If we take the Laplace transform of Equations (3 and 5)

(69) $$\frac{\partial^{\beta }n\left(x,t\right)}{\partial t^{\beta }}=-\frac{\partial J_{\beta }\left(x,t\right)}{\partial x}$$

(70) $$J_{\beta }\left(x,t\right)=-D_{\beta }\frac{\partial n\left(x,t\right)}{\partial x}$$

The equations of the diffusion model are

(71) $$\frac{\partial \Delta V\left(x,t\right)}{\partial x}=-\frac{1}{D_{\beta }c_{\mu }}i_{\beta }\left(x,t\right)$$

(72) $$\frac{\partial \Delta i_{\beta }\left(x,t\right)}{\partial x}=-c_{\mu }s^{\beta }\Delta V\left(x,t\right)$$

Passing to the regular current, we have

(73) $$\frac{\partial \Delta V\left(x,t\right)}{\partial x}=-\frac{\omega_{d}^{\beta -1}}{D_{\beta }c_{\mu }}\Delta i_{d}\left(x,t\right)$$

(74) $$\frac{\partial \Delta i_{d}\left(x,t\right)}{\partial x}=-c_{\mu }\omega_{d}^{1-\beta }s^{\beta }\Delta i_{d}\left(x,t\right)$$

By comparing with (67, 68), we obtain

(75) $$\chi_{1}=\frac{R_{d}}{L}$$

(76) $$\zeta_{3}=\frac{Q_{\beta }}{L}s^{\beta }$$

We define a distributed resistance

(77) $$r_{1}=\frac{R_{d}}{L}$$

and a distributed CPE parameter

(78) $$q_{3}=\frac{Q_{\beta }}{L}$$

This is the model of Figure 3a, with an added series resistance.

## Appendix 2. Transient Current After a Voltage Step

We search for the solution of the current under a voltage step *V*_0_ with zero initial conditions

(79) $$V\left(x,0\right)=0,0<x<L$$

and Equation (13). From the Caputo time‐fractional derivative, with a Laplace transform of

(80) $$L\left[\frac{\partial^{\beta }V\left(x,t\right)}{\partial t^{\beta }}\right]=s^{\beta }v\left(x,s\right)-s^{\beta -1}v\left(x,0^{+}\right)$$

and the finite Fourier sine transform method,^[^ ^62^ ^]^ it can be shown that the resulting voltage distribution is

(81) $$\frac{V\left(x,t\right)}{V_{0}}=1-2\sum_{k=1}^{\infty }\alpha_{k}^{-1}\sin\left(\alpha_{k}\frac{x}{L}\right)E_{\beta }\left[-\alpha_{k}^{2}{\left(\omega_{d}\;t\right)}^{\beta }\right]$$

where α_*k*_ = π(*k* − 1/2) with k=1,2,… ^[^ ^63^ ^]^ and *E*_β_(−*z*) denotes the one‐parameter Mittag‐Leffler function^[^ ^48^ ^]^ defined by

(82) $$E_{\beta }\left(-z\right)=\sum_{k=0}^{\infty }\frac{{\left(-z\right)}^{k}}{\Gamma \left(\beta k+1\right)},\;\beta >0$$

For the case β  =  1, *E*_1_(−*z*) = *e* ^−*z*^.

The measurable current transient, at the injection point *x*  =  0, can be obtained by differentiation of Equation (81) with the use of the relation^[^ ^64^ ^]^

(83) $$I_{d}=i\left(0,t\right)=-\frac{L}{R_{d}}\left({\left.\frac{\partial V\left(x,t\right)}{\partial x}\right|}_{x=0}\right)$$

yielding

(84) $$I_{d}\left(t\right)=\frac{2V_{0}}{R_{d}}\sum_{k=1}^{\infty }E_{\beta }\left[-\alpha_{k}^{2}{\left(\omega_{d}\;t\right)}^{\beta }\right]$$

Similar results have been recently obtained by considering a galvanostatic charging mode.^[^ ^65^ ^]^

## Conflict of Interest

The authors declare no conflict of interest.

## Supporting information



Supporting Information

## Data Availability

The data presented here can be accessed at https://doi.org/10.5281/zenodo.15272857 (Zenodo) under the license CC BY 4.0 (Creative Commons Attribution 4.0 International).
