# Supplementary material for: Transient Charging of Mixed Ionic‐Electronic Conductors by Anomalous Diffusion
Source: Adv Mater. 2025 Aug 23;38(51):e07739. doi: 10.1002/adma.202507739 (PMC13569026; doi:10.1002/adma.202507739)
Supplement: Supplementary file 1 [file ADMA-38-e07739-s001.docx]

Supporting Information

# Transient Charging of Mixed Ionic-Electronic Conductors by Anomalous Diffusion

**Heyi Zhang,^1,2^, Gonzalo Rivera-Sierra,^1^** **Shirin Siahjani-Gultekin,^1,3^ Jenifer Rubio-Magnieto,^1^ Anis Allagui,^4^ Ignacio Sanjuán,^2^ David Franco,^2^ Antonio Guerrero,^2^* Enrique H. Balaguera,^5^* Juan Bisquert^1^***

^1^Instituto de Tecnología Química (ITQ). Universitat Politècnica de València- Consejo Superior de Investigaciones Científicas (UPV-CSIC). 46022 València, Spain

^2^Institute of Advanced Materials (INAM), Universitat Jaume I, 12006 Castelló, Spain

^3^ Solar Energy Institute, Ege University, 35100, Izmir, Türkiye

^4^ Department of Sustainable and Renewable Energy Engineering, University of Sharjah, PO Box 27272, Sharjah, United Arab Emirates

^5^Escuela Superior de Ciencias Experimentales y Tecnología (ESCET), Universidad Rey Juan Carlos, 28933 Móstoles, Madrid, Spain

*Corresponding authors e-mail: [aguerrer@uji.es](mailto:aguerrer@uji.es) [enrique.hernandez@urjc.es](mailto:enrique.hernandez@urjc.es), [jbisquer@itq.upv.es](mailto:jbisquer@itq.upv.es)

# Extracted Parameters Lists of Impedance Fitting

Table S1. Extracted parameters of devices obtained by fitting with the DX Type 12 – Bisquert t#3 model.

| Device | $L (nm)$ | $R_{s} (\Omega)$ | $R_{d} (k\Omega)$ | $Q_{d} ({10}^{-4} F s^{\beta-1})$ | $\beta$ | $\omega_{d} (rad/s)$ | $D_{\beta} ({10}^{-9} {cm}^{2} s^{-\beta})$ |
| --- | --- | --- | --- | --- | --- | --- | --- |
| P630 | 630 | 924 | 25.8 | 12.68 | 0.801 | 15.5 | 35.5 |
| P200 | 200 | 926 | 17.0 | 3.53 | 0.811 | 63.2 | 11.5 |
| P120 | 120 | 948 | 26.1 | 1.91 | 0.849 | 90.1 | 6.59 |
| P090 | 90 | 961 | 23.4 | 1.29 | 0.904 | 190 | 9.27 |
| WO_3_ a | 4740 | 619 | 144 | 0.679 | 0.817 | 43.9 | 4930 |
| WO_3_ b | 3540 | 550 | 375 | 0.550 | 0.819 | 24.1 | 1700 |
| n-PBDF | 21 | 126 | 881 | 2.12 | 0.717 | 3.33 | 1.04×10⁻² |

Note:

The thickness (L) for all PEDOT:PSS films was determined using step-height measurements via contact-mode AFM.

The parameters corresponding to the WO₃ b sample are discussed in detail in the main manuscript due to their representative character and significance in model fitting.

The spin-coating process of PEDOT:PSS films is as follows:

P630, spin-coated at 300 rpm for 55s, then 1000 rpm for 5 seconds for spreading the liquid film; P200, P120, and P090, correspondingly, at 1000, 2500, and 4000 rpm, for 60 seconds.

Table S2. Extracted chemical capacitance parameters $C_{\mu}^{*}$ and $c_{\mu}$.

| Device | $C_{\mu}^{*} ({10}^{-4} F)$ | $c_{\mu} (F {cm}^{-3})$ |
| --- | --- | --- |
| P630 | 30.2 | 228.5 |
| P200 | 5.4 | 127.6 |
| P120 | 2.5 | 101.0 |
| P090 | 1.5 | 76.6 |
| WO_3_ a | 1.1 | 1.1 |
| WO_3_ b | 1.1 | 1.4 |
| n-PBDF | 16.7 | 1013.8 |

Note:

The effective chemical capacitance $C_{\mu}^{*}$ and the specific volumetric capacitance $c_{\mu}$ were calculated from the fitted $R_{d}$, $Q_{d}$, and $\beta$ parameters in Table S1 by Eq. (23). Area values used: 0.21 cm² for PEDOT:PSS and WO₃ devices, and 0.785 cm² for n-PBDF.

# Supporting data of PEDOT:PSS system

Table S3. Root-mean-square Roughness (Rq) and Arithmetic-average Roughness (Ra) obtained from contact AFM measurement.

| Sample | Rq (nm) | Ra (nm) |
| --- | --- | --- |
| P630 | 2.61 | 2.08 |
| P200 | 2.15 | 1.70 |
| P120 | 1.76 | 1.35 |
| P090 | 1.48 | 1.17 |

Figure S1. (a). Nyquist plot of the impedance data of different thickness samples under applied potential 0.5 V with frequency range from 10^5^ Hz to 0.1 Hz; (b) High frequency range of the impedance data showing the transmission line part; (c) Logarithmic plot curve (with linear slopes in red) of the transient current response under a step voltage at initially V_I_=0.3 V and ∆V=0.2 V as indicated.

Figure S2. Bode plots of impedance data for PEDOT:PSS films (P090–P630) displaying the magnitude |Z| and phase angle versus logarithmic frequency.

Figure S3. Comparison of the normalized $\omega_{d}$ value extracted from impedance and transient decay for PEDOT:PSS films with varying thickness. While slight differences in absolute values are observed between the two methods, the thickness-dependent trend is consistent, demonstrating the robustness of the extracted diffusion timescales across measurement domains.

Following the discussion presented in the main text, we observe that the diffusion coefficients can be directly correlated with the diffusion frequencies extracted from either impedance spectroscopy fits or transient charge accumulation analysis. However, in cases where capacitive effects, such as those discussed in Section 2.4 (Transmission Line Model), occur within frequency ranges overlapping with that of the transmission line (and hence the diffusion process), the transient charge measurements may be affected. This is particularly relevant in practical devices, where multiple interfacial and bulk processes coexist and influence each other, leading to mutual interference within the same time domain.

In the WO₃-based system, the frequency range in which $\omega_{d}$ is located is consistent across both analysis methods, suggesting minimal interference from secondary effects. In contrast, for the other two systems, a systematic shift in the transient-derived $\omega_{d}$ is observed: increased values in the case of PEDOT:PSS and decreased values for n-PBDF, when compared to those obtained from impedance spectroscopy. To verify that these shifts are consistent across different film thicknesses, the normalized values of $\omega_{d}$ from both methods for PEDOT:PSS devices are plotted in Fig. S3, revealing that although absolute values differ, the same monotonic trend is preserved, confirming a systematic offset rather than random error. This confirms that the shift is systematic and likely originates from one of the secondary effects discussed in the main text.

For the n-PBDF system, the active layer is significantly thinner, on the order of 10 nm, which makes it more susceptible to substrate-related capacitive effects. These can become active within the same frequency range as diffusion and artificially shift the extracted $\omega_{d}$, typically increasing its value. On the other hand, in the PEDOT:PSS system, the effect is attributed to contact: while WO₃ and n-PBDF devices employ ITO contacts, PEDOT:PSS is contacted with Au, which introduces stronger capacitive contributions at the MIEC/contact interface, likely responsible for the observed upward shift in $\omega_{d}$ in transient measurements.

Figure S4. AFM topography images of PEDOT:PSS films (P090–P630) deposited by spin-coating under controlled conditions. The images demonstrate systematic surface morphology evolution with increasing film thickness. Root-mean-square roughness (Rq) and arithmetic-average roughness (Ra) values are listed in Table S3.

Figure S5. (a). Photographs of the substrates used for PEDOT:PSS films-electrolyte vertical setup used for measurements, and (b) the electrolyte well configuration and vertical two-terminal assembly.

# Supporting data of WO_3_ system

Figure S6. (a) Nyquist plot of impedance data for a WO₃ thin film in a two-terminal vertical configuration. Measurement performed at -0.3 V vs Ag/AgCl in 0.1 M H_2_SO_4_ aqueous electrolyte over 10⁵ to 1 Hz. Black symbols represent experimental data, and red solid lines indicate fitted curves using the fractional transmission-line model; (b) Corresponding transient current decay curve under a –0.2V voltage step, plotted in log-log scale. The fitted linear regions are used to extract the fractional exponent β; (c) Bode plot (magnitude |Z| and phase angle versus log frequency) for a WO₃ film with thickness 4.7 and 3.5 um; (d) Bode plot for WO₃ films.

Figure S7. Top-view SEM image of the surface morphology of the WO₃ thin films.

Figure S8. Photographs showing the electrochromic color change of a WO₃ thin film upon electrochemical reduction. The image on the left displays the bleached (transparent) state at open-circuit potential, while the image on the right shows the colored (blue) state after applying a negative potential of –0.3 V vs Ag/AgCl in 0.1 M H2SO4 aqueous electrolyte. The uniform color transition indicates homogeneous ionic insertion throughout the film, confirming its suitability for confined vertical ionic transport studies.

Figure S9. Electrochemical impedance and transient response of a WO₃ thin film measured in a three-electrode configuration. A coiled platinum (Pt) wire was used as the counter electrode (CE), an Ag/AgCl electrode as the reference electrode (RE), and the WO₃/ITO film as the working electrode (WE). All electrodes were immersed in 0.1 M H₂SO₄ aqueous electrolyte at room temperature. (a) Nyquist impedance spectrum of the WO₃ thin film acquired over the frequency range 10⁵ to 1 Hz at an applied potential of –0.3 V vs Ag/AgCl; (b) Corresponding transient current decay curve under a -0.2V potential step, plotted in log-log scale. The power-law decay behavior is observed. The results confirm that anomalous ionic diffusion behavior observed in two-terminal devices persists under three-electrode electrochemical control.

# Supporting data of n-PBDF system

Figure S10. (a) Nyquist impedance spectrum of an n-doped PBDF thin film measured in a two-terminal vertical configuration at 0.3 V vs Ag/AgCl in 0.1 M NaCl aqueous electrolyte, frequency range 10⁵–0.1 Hz; (b) Transient current decay curve under a 0.2V potential step. The log-log plot highlights two distinct power-law regions; (c) Bode plots for two n-PBDF films. The thickness of the 2000 rpm sample is 43 nm, and the 4000 rpm sample is discussed in the paper.
